# Supplementary material for: Human kinesin-5 KIF11 drives the helical motion of anti-parallel and parallel microtubules around each other
Source: EMBO J. 2024 Feb 29;43(7):1244–56. doi: 10.1038/s44318-024-00048-x (PMC10987665; doi:10.1038/s44318-024-00048-x)
Supplement: Supplementary file 1 — Appendix [file 44318_2024_48_MOESM1_ESM.pdf]

Human kinesin-5 KIF11 drives the helical motion of anti-parallel and parallel microtubules around each other

Laura Meißner, Lukas Niese, Irene Schüring, Aniruddha Mitra, Stefan Diez

---

Contents

**Appendix Figure S1:**	SDS gel analysis of KIF11-EGFP purification	2
**Appendix Figure S2:**	KIF11 drives helical motion with and without EGFP tag	2
**Appendix Figure S3:**	Motility parameters as function of cargo microtubule length and particular fixed microtubules	3
**Appendix Figure S4:**	KIF11 concentration does not influence motility parameters considerably	4
**Appendix Figure S5:**	Buffer composition does not influence motility parameters of KIF11 considerably	5
**Appendix Figure S6:**	Motility modes of KIF11-driven microtubule-microtubule sliding	6
**Appendix Table S1:**	Breakdown of data sets	7
**Appendix Table S2:**	Primers used for cloning	7

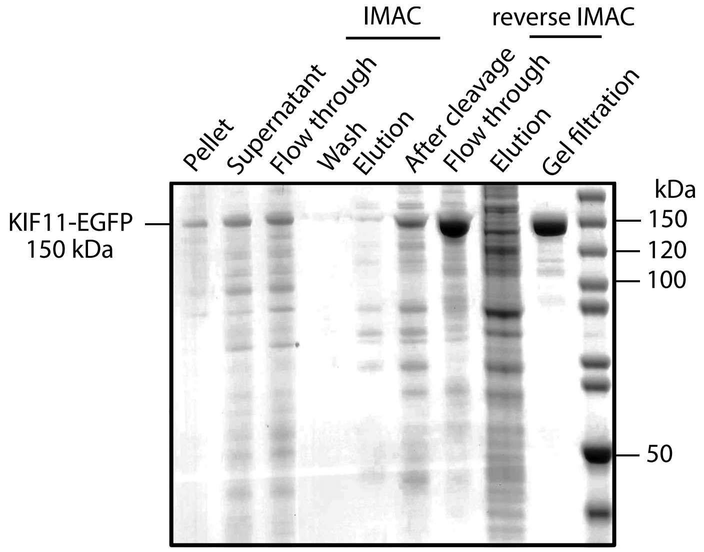

Appendix Figure S1: SDS gel analysis of KIF11-EGFP purification. KIF11-EGFP was expressed in SF9 cells and samples were taken for the individual purification steps. Lysate was separated in insoluble (pellet) and soluble fraction (supernatant). The supernatant was applied to a HiTrap column (immobilized metal affinity chromatography, IMAC). Proteins without His_6 tag did not bind to the column (flow through) and KIF11-EGFP was eluted with imidazole. After tag cleavage, the protein solution was re-applied to the column, did not bind to it (reverse IMAC, flow through) and mostly unspecific proteins were eluted with imidazole. In the last step, the protein solution was subjected to a gel filtration.

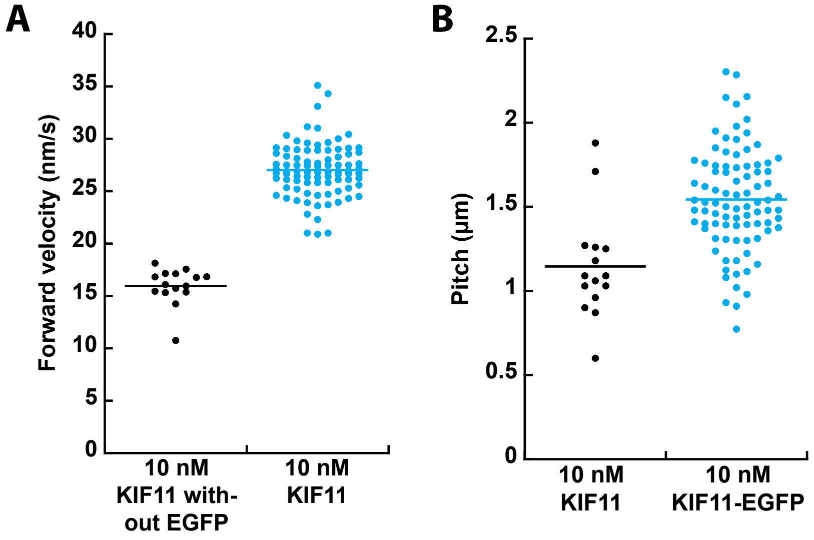

Appendix Figure S2: KIF11 drives helical motion with and without EGFP tag. Comparison of forward velocity (A) and pitch (B) of cargo microtubules driven by KIF11 with and without a C-terminal EGFP tag. *KIF11 without EGFP tag:* forward velocity of 16.0 ± 1.8 nm/s and pitch of 1.15 ± 0.32 µm, n = 15. *KIF11 with EGFP tag:* Data from Fig. 1E-G in the main text, n = 88.

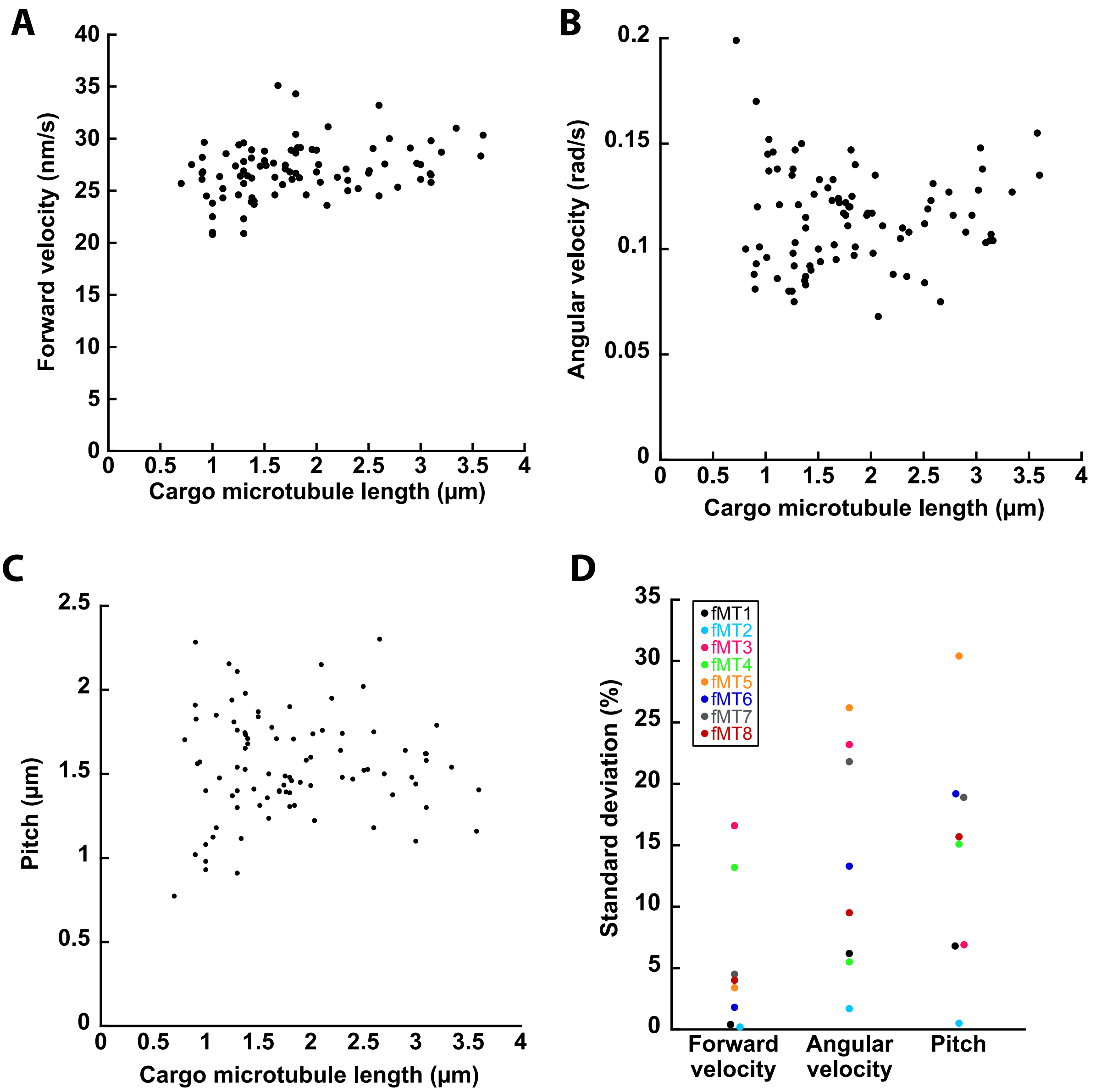

Appendix Figure S3: Motility parameters as function of cargo microtubule length and particular fixed microtubules. **A**) Forward velocity, **B**) angular velocity, and **C**) pitch as function of cargo microtubule length. **D**) Standard deviations of the motility parameters (in %) for cargo microtubules sliding along particular fixed microtubules (fMT1 - fMT8, color-coded).

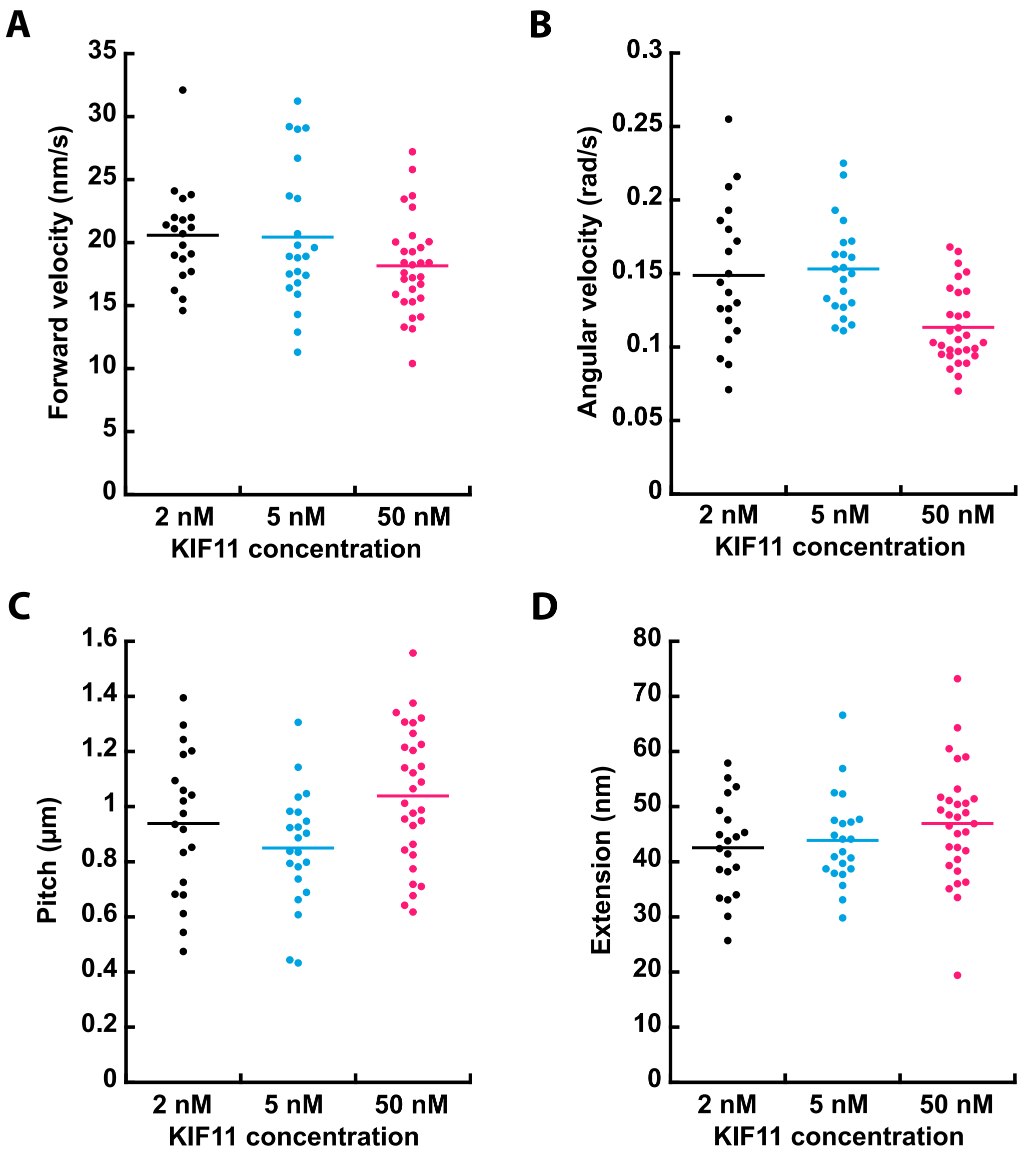

Appendix Figure S4: KIF11 concentration does not influence motility parameters considerably. Comparison of forward velocity (A), angular velocity (B), pitch (C) and extension (D) of cargo microtubules driven by KIF11 in different concentrations. 2 nM KIF11: 20.6 ± 3.8 nm/s, 0.149 ± 0.048 rad/s, 0.94 ± 0.26 µm, 42.5 ± 8.7 nm, n = 20; 5 nM KIF11: 20.4 ± 5.6 nm/s, 0.153 ± 0.032 rad/s, 0.85 ± 0.21 µm, 43.9 ± 8.3 nm, n = 22; 50 nM KIF11: 18.1 ± 3.8 nm/s, 0.113 ± 0.026 rad/s, 1.04 ± 0.25 µm, 47.0 ± 10.4 nm, n = 30.

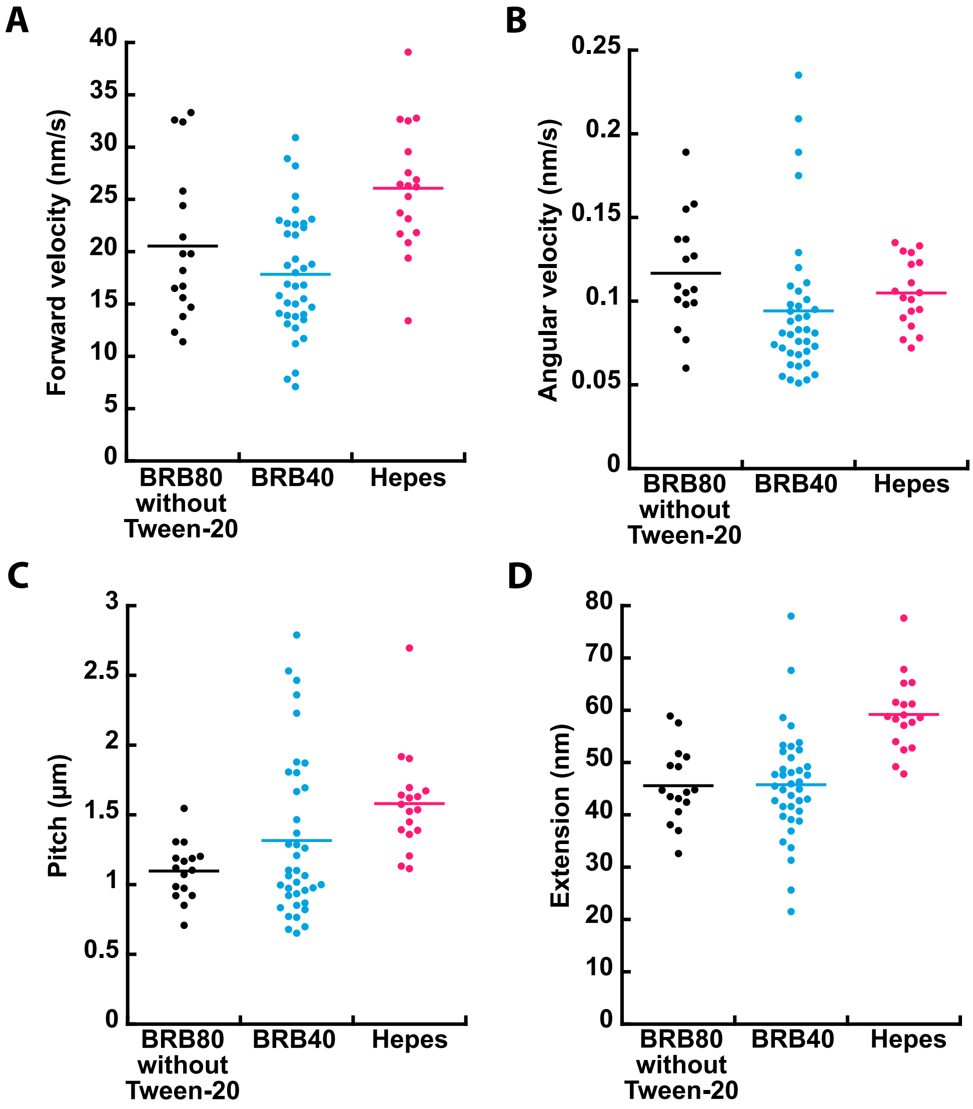

Appendix Figure S5: Buffer composition does not influence motility parameters of KIF11 considerably. Comparison of forward velocity (A), angular velocity (B), pitch (C) and extension (D) of cargo microtubules driven by KIF11 in different motility buffers. BRB80 without Tween-20: 20.5 ± 7.2 nm/s, 0.117 ± 0.033 rad/s, 1.10 ± 0.20 µm, 45.6 ± 7.1 nm, n = 16; BRB40: 17.8 ± 5.8 nm/s, 0.094 ± 0.043 rad/s, 1.32 ± 0.57 µm, 45.8 ± 10.3 nm, n = 38; 20 mM Hepes with 50 mM KCl: 26.1 ± 5.9 nm/s, 0.105 ± 0.020 rad/s, 1.58 ± 0.36 µm, 59.2 ± 7.1 nm, n = 18.

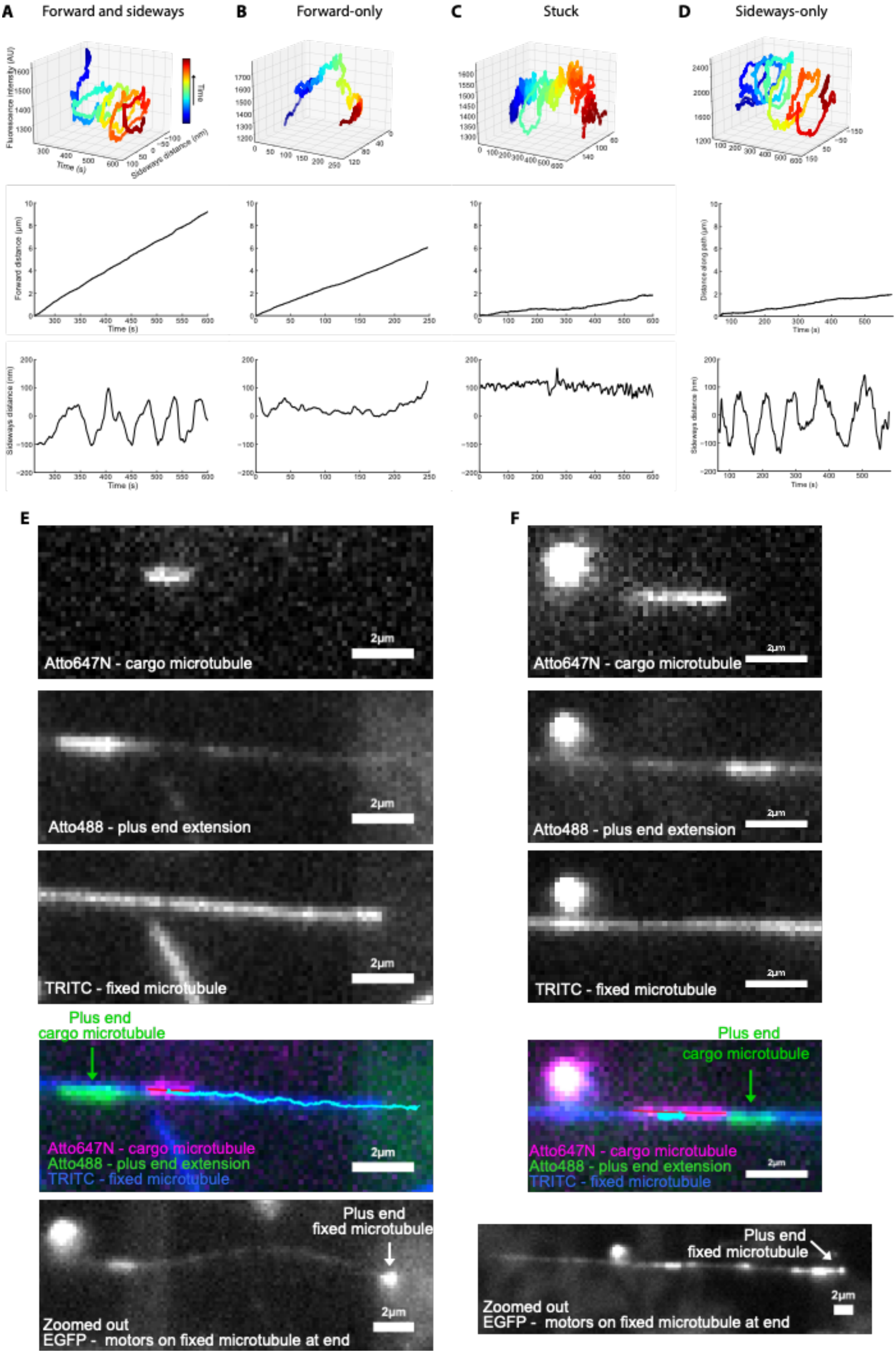
A
Forward and sideways
B
Forward-only
C
Stuck
D
Sideways-only
Fluorescence intensity (AU)
Time
Time (s)
Sideways distance (nm)
Forward distance (µm)
Distance along path (µm)
Sideways distance (nm)
E
Atto647N - cargo microtubule
Atto488 - plus end extension
TRITC - fixed microtubule
Plus end
cargo microtubule
Atto647N - cargo microtubule
Atto488 - plus end extension
TRITC - fixed microtubule
Plus end
fixed microtubule
Zoomed out
EGFP - motors on fixed microtubule at end
2µm
F
Atto647N - cargo microtubule
Atto488 - plus end extension
TRITC - fixed microtubule
Plus end
cargo microtubule
Atto647N - cargo microtubule
Atto488 - plus end extension
TRITC - fixed microtubule
Plus end
fixed microtubule
Zoomed out
EGFP - motors on fixed microtubule at end
2µm

Appendix Figure S6: Motility modes of KIF11-driven microtubule-microtubule sliding. Cargo microtubules **A**) moved forward and sideways, **B**) moved only forward, **C**) were stuck or **D**) moved only sideways (top row: 3D analysis; middle row: forward displacement; bottom row: sideways displacement). **E**) Forward and sideways event: fluorescence micrographs of individual channels, merge and EGFP signal for determination of microtubule polarity (bottom). Fixed (plus end on right side) and cargo microtubule (plus end on left side) are anti-parallel. Data also shown in Movie EV3. **F**) Sideways-only event: fluorescence micrographs of individual channels, merge and EGFP signal for determination of microtubule polarity (bottom). Fixed (plus end on right side) and cargo microtubule (plus end on right side) are parallel. Data also shown in Movie EV4.

Appendix Table S1: Breakdown of data sets. The data sets can be broken down into the following number of events per category.

**Condition**	Total number of observed cargo microtubules	Cargo microtubules **immobile in forward direction**	Cargo microtubules **mobile in forward direction**	**Forward-and-sideways** cargo microtubules	**Sideways-only** cargo microtubules
10 nM KIF11	647	341	306	88	161
NL14	328	209	119	12	1
NL16	553	342	211	11	5
NL20	322	134	188	18	15
NL22	340	198	142	7	1
All NL mutants pooled	1543	883	660	48	22

Appendix Table S2: Primers used for cloning

**Construct**	**Primers**
KIF11	AATAATAACAT*GCGGCCGC*AATGGCGTCGCAGCCAAATTCG<br>AATAATAACAT*GGCGCGCC*AAGGTTGATCTGGGCTCGCAG
KIF11-EGFP	Same as KIF11
KIF11-EGFP NL14	ATTAAGGAGTATACGGAGGAGATAGA<br>GGTGAGTTTCTGATTCACTTCAGG
KIF11-EGFP NL16	ATTAAGGAGTATACGGAGGAGATAGA<br>TTTTTTGGTGAGTTTCTGATTCACTT
KIF11-EGFP NL20	GGTTCCATTAAGGAGTATACGGAGGAGATAGAA<br>AAGAGCTTTTTTGGTGAGTTTCTG
KIF11-EGFP NL22	GGTTCCGGCTCCATTAAGGAGTATACGGAGGAGATAGA<br>AAGAGCTTTTTTGGTGAGTTTCTG
